# Supplementary material for: Development of a computer-aided design software for the quantitative evaluation of aesthetic damage
Source: PLoS One. 2019 Dec 18;14(12):e0226322. doi: 10.1371/journal.pone.0226322 (PMC6919621; doi:10.1371/journal.pone.0226322)
Supplement: S1 Code site — 1. (ZIP) [file pone.0226322.s001.zip › [EESC_jr][Projeto]Relatorio_07_21/reports/templates_zip/gentelella-master.zip!/gentelella-master/vendors/DateJS/reports/lcov-report/core/parsing_grammar.js.html]

Code coverage report for core/parsing\_grammar.js


# Code coverage report for core/parsing\_grammar.js

## Statements: 91.59% (98 / 107)      Branches: 56.25% (9 / 16)      Functions: 96.55% (28 / 29)      Lines: 91.59% (98 / 107)      Ignored: none

All files » core/ » parsing\_grammar.js

```
|  |  |  |
| --- | --- | --- |
| 1 2 3 4 5 6 7 8 9 10 11 12 13 14 15 16 17 18 19 20 21 22 23 24 25 26 27 28 29 30 31 32 33 34 35 36 37 38 39 40 41 42 43 44 45 46 47 48 49 50 51 52 53 54 55 56 57 58 59 60 61 62 63 64 65 66 67 68 69 70 71 72 73 74 75 76 77 78 79 80 81 82 83 84 85 86 87 88 89 90 91 92 93 94 95 96 97 98 99 100 101 102 103 104 105 106 107 108 109 110 111 112 113 114 115 116 117 118 119 120 121 122 123 124 125 126 127 128 129 130 131 132 133 134 135 136 137 138 139 140 141 142 143 144 145 146 147 148 149 150 151 152 153 154 155 156 157 158 159 160 161 162 163 164 165 166 167 168 169 170 171 172 173 174 175 176 177 178 179 180 181 182 183 184 185 186 187 188 189 190 191 192 193 194 195 196 197 198 199 200 201 202 203 204 205 206 207 208 209 210 211 212 213 214 215 216 217 218 219 220 221 222 223 224 225 226 227 228 229 230 231 232 233 234 235 236 237 238 239 240 241 242 243 244 245 246 247 248 249 250 251 252 253 254 255 256 257 258 259 260 261 262 263 264 265 266 267 268 269 270 271 272 273 274 275 276 277 278 279 280 281 282 283 284 285 286 287 288 289 290 291 292 293 294 295 296 297 298 299 300 301 302 303 304 305 306 307 308 309 310 | 1 1 1 1   1 480     1 1 1 1   1 1 960 960 960 960 960 5440   960   960   1 1280   1 3040 320   2720     1 480   1   1 20 20     1                       1 1 1 1 20   1           1   160                                                                                 160 1760     160   160 160 160   160 160       160 480   160                                                                     160 1280     160 160   13 10         160 160 160   160 160 160   160 530         160   6 6         160   451 451       160 160   449 449             1   160   160 160 160     160   456 451       160   160           91 91                 56             20         160       1         1                                                 1 530 530 47       530 | ``` (function () { 	var $D = Date; 	$D.Grammar = {}; 	var _ = $D.Parsing.Operators, g = $D.Grammar, t = $D.Translator, _fn; 	// Allow rolling up into general purpose rules 	_fn = function () { 		return _.each(_.any.apply(null, arguments), _.not(g.ctoken2("timeContext"))); 	}; 	 	g.datePartDelimiter = _.rtoken(/^([\s\-\.\,\/\x27]+)/); 	g.timePartDelimiter = _.stoken(":"); 	g.whiteSpace = _.rtoken(/^\s*/); 	g.generalDelimiter = _.rtoken(/^(([\s\,]\|at\|@\|on)+)/);    	var _C = {}; 	g.ctoken = function (keys) { 		var fn = _C[keys]; 		Eif (! fn) { 			var c = Date.CultureInfo.regexPatterns; 			var kx = keys.split(/\s+/), px = []; 			for (var i = 0; i < kx.length ; i++) { 				px.push(_.replace(_.rtoken(c[kx[i]]), kx[i])); 			} 			fn = _C[keys] = _.any.apply(null, px); 		} 		return fn; 	}; 	g.ctoken2 = function (key) { 		return _.rtoken(Date.CultureInfo.regexPatterns[key]); 	}; 	var cacheProcessRtoken = function (key, token, type, eachToken) { 		if (eachToken) { 			g[key] = _.cache(_.process(_.each(_.rtoken(token),_.optional(g.ctoken2(eachToken))), type)); 		} else { 			g[key] = _.cache(_.process(_.rtoken(token), type)); 		} 	}; 	var cacheProcessCtoken = function (token, type) { 		return _.cache(_.process(g.ctoken2(token), type)); 	}; 	var _F = {}; //function cache   	var _get = function (f) { 		_F[f] = (_F[f] \|\| g.format(f)[0]); 		return _F[f]; 	};   	g.allformats = function (fx) { 		var rx = []; 		if (fx instanceof Array) { 			for (var i = 0; i < fx.length; i++) { 				rx.push(_get(fx[i])); 			} 		} else { 			rx.push(_get(fx)); 		} 		return rx; 	};    	g.formats = function (fx) { 		Eif (fx instanceof Array) { 			var rx = []; 			for (var i = 0 ; i < fx.length ; i++) { 				rx.push(_get(fx[i])); 			} 			return _.any.apply(null, rx); 		} else { 			return _get(fx); 		} 	};   	var grammarFormats = { 		 timeFormats: function(){ 			var i, 			RTokenKeys = [ 				"h", 				"hh", 				"H", 				"HH", 				"m", 				"mm", 				"s", 				"ss", 				"ss.s", 				"z", 				"zz" 			], 			RToken = [ 				/^(0[0-9]\|1[0-2]\|[1-9])/, 				/^(0[0-9]\|1[0-2])/, 				/^([0-1][0-9]\|2[0-3]\|[0-9])/, 				/^([0-1][0-9]\|2[0-3])/, 				/^([0-5][0-9]\|[0-9])/, 				/^[0-5][0-9]/, 				/^([0-5][0-9]\|[0-9])/, 				/^[0-5][0-9]/, 				/^[0-5][0-9]\.[0-9]{1,3}/, 				/^((\+\|\-)\s*\d\d\d\d)\|((\+\|\-)\d\d\:?\d\d)/, 				/^((\+\|\-)\s*\d\d\d\d)\|((\+\|\-)\d\d\:?\d\d)/ 			], 			tokens = [ 				t.hour, 				t.hour, 				t.hour, 				t.minute, 				t.minute, 				t.second, 				t.second, 				t.secondAndMillisecond, 				t.timezone, 				t.timezone, 				t.timezone 			];   			for (i=0; i < RTokenKeys.length; i++) { 				cacheProcessRtoken(RTokenKeys[i], RToken[i], tokens[i]); 			}   			g.hms = _.cache(_.sequence([g.H, g.m, g.s], g.timePartDelimiter));   			g.t = cacheProcessCtoken("shortMeridian", t.meridian); 			g.tt = cacheProcessCtoken("longMeridian", t.meridian); 			g.zzz = cacheProcessCtoken("timezone", t.timezone);   			g.timeSuffix = _.each(_.ignore(g.whiteSpace), _.set([ g.tt, g.zzz ])); 			g.time = _.each(_.optional(_.ignore(_.stoken("T"))), g.hms, g.timeSuffix); 		 }, 		 dateFormats: function () { 			// pre-loaded rules for different date part order preferences 			var _setfn = function () { 				return  _.set(arguments, g.datePartDelimiter); 			}; 			var i, 			RTokenKeys = [ 				"d", 				"dd", 				"M", 				"MM", 				"y", 				"yy", 				"yyy", 				"yyyy" 			], 			RToken = [ 				/^([0-2]\d\|3[0-1]\|\d)/, 				/^([0-2]\d\|3[0-1])/, 				/^(1[0-2]\|0\d\|\d)/, 				/^(1[0-2]\|0\d)/, 				/^(\d+)/, 				/^(\d\d)/, 				/^(\d\d?\d?\d?)/, 				/^(\d\d\d\d)/ 			], 			tokens = [ 				t.day, 				t.day, 				t.month, 				t.month, 				t.year, 				t.year, 				t.year, 				t.year 			], 			eachToken = [ 				"ordinalSuffix", 				"ordinalSuffix" 			]; 			for (i=0; i < RTokenKeys.length; i++) { 				cacheProcessRtoken(RTokenKeys[i], RToken[i], tokens[i], eachToken[i]); 			}   			g.MMM = g.MMMM = _.cache(_.process(g.ctoken("jan feb mar apr may jun jul aug sep oct nov dec"), t.month)); 			g.ddd = g.dddd = _.cache(_.process(g.ctoken("sun mon tue wed thu fri sat"), 				function (s) { 					return function () { 						this.weekday = s; 					}; 				} 			));   			g.day = _fn(g.d, g.dd); 			g.month = _fn(g.M, g.MMM); 			g.year = _fn(g.yyyy, g.yy);   			g.mdy = _setfn(g.ddd, g.month, g.day, g.year); 			g.ymd = _setfn(g.ddd, g.year, g.month, g.day); 			g.dmy = _setfn(g.ddd, g.day, g.month, g.year); 						 			g.date = function (s) { 				return ((g[Date.CultureInfo.dateElementOrder] \|\| g.mdy).call(this, s)); 			}; 		 }, 		 relative: function () { 			// relative date / time expressions 			g.orientation = _.process(g.ctoken("past future"), 				function (s) { 					return function () { 						this.orient = s; 					}; 				} 			);   			g.operator = _.process(g.ctoken("add subtract"), 				function (s) { 					return function () { 						this.operator = s; 					}; 				} 			); 			g.rday = _.process(g.ctoken("yesterday tomorrow today now"), t.rday); 			g.unit = _.process(g.ctoken("second minute hour day week month year"), 				function (s) { 					return function () { 						this.unit = s; 					}; 				} 			); 		 } 	};   	g.buildGrammarFormats = function () { 		// these need to be rebuilt every time the language changes. 		_C = {};   		grammarFormats.timeFormats(); 		grammarFormats.dateFormats(); 		grammarFormats.relative();   		 		g.value = _.process(_.rtoken(/^([-+]?\d+)?(st\|nd\|rd\|th)?/), 			function (s) { 				return function () { 					this.value = s.replace(/\D/g, ""); 				}; 			} 		); 		g.expression = _.set([g.rday, g.operator, g.value, g.unit, g.orientation, g.ddd, g.MMM ]);   		g.format = _.process(_.many( 			_.any( 				// translate format specifiers into grammar rules 				_.process( 					_.rtoken(/^(dd?d?d?(?!e)\|MM?M?M?\|yy?y?y?\|hh?\|HH?\|mm?\|ss?\|tt?\|zz?z?)/), 						function (fmt) { 							Eif (g[fmt]) { 								return g[fmt]; 							} else { 								throw $D.Parsing.Exception(fmt); 							} 						} 					), 					// translate separator tokens into token rules 					_.process(_.rtoken(/^[^dMyhHmstz]+/), // all legal separators  						function (s) { 							return _.ignore(_.stoken(s)); 						} 					) 				) 			), 			// construct the parser ... 			function (rules) { 				return _.process(_.each.apply(null, rules), t.finishExact); 			} 		);   		// starting rule for general purpose grammar 		g._start = _.process(_.set([ g.date, g.time, g.expression ], 		g.generalDelimiter, g.whiteSpace), t.finish); 	};   	g.buildGrammarFormats(); 	// parsing date format specifiers - ex: "h:m:s tt"  	// this little guy will generate a custom parser based 	// on the format string, ex: g.format("h:m:s tt") 	// check for these formats first 	g._formats = g.formats([ 		"\"yyyy-MM-ddTHH:mm:ssZ\"", 		"yyyy-MM-ddTHH:mm:ss.sz", 		"yyyy-MM-ddTHH:mm:ssZ", 		"yyyy-MM-ddTHH:mm:ssz", 		"yyyy-MM-ddTHH:mm:ss", 		"yyyy-MM-ddTHH:mmZ", 		"yyyy-MM-ddTHH:mmz", 		"yyyy-MM-ddTHH:mm", 		"ddd, MMM dd, yyyy H:mm:ss tt", 		"ddd MMM d yyyy HH:mm:ss zzz", 		"MMddyyyy", 		"ddMMyyyy", 		"Mddyyyy", 		"ddMyyyy", 		"Mdyyyy", 		"dMyyyy", 		"yyyy", 		"Mdyy", 		"dMyy", 		"d" 	]); 	 	// real starting rule: tries selected formats first,  	// then general purpose rule 	g.start = function (s) { 		try { 			var r = g._formats.call({}, s); 			Iif (r[1].length === 0) { 				return r; 			} 		} catch (e) {} 		return g._start.call({}, s); 	}; }()); ``` |
```

Generated by istanbul at Sun Nov 16 2014 12:08:33 GMT-0800 (PST)
